# Endocytic Pathways Used by Andes Virus to Enter Primary Human Lung Endothelial Cells

**DOI:** 10.1371/journal.pone.0164768

**Published:** 2016-10-25

**Authors:** Cheng-Feng Chiang, Mike Flint, Jin-Mann S. Lin, Christina F. Spiropoulou

**Affiliations:** 1 Viral Special Pathogens Branch, Division of High-Consequence Pathogens and Pathology, Centers for Disease Control and Prevention, Atlanta, Georgia, United States of America; 2 Chronic Viral Diseases Branch, Division of High-Consequence Pathogens and Pathology, Centers for Disease Control and Prevention, Atlanta, Georgia, United States of America; Division of Clinical Research, UNITED STATES

## Abstract

Andes virus (ANDV) is the major cause of hantavirus pulmonary syndrome (HPS) in South America. Despite a high fatality rate (up to 40%), no vaccines or antiviral therapies are approved to treat ANDV infection. To understand the role of endocytic pathways in ANDV infection, we used 3 complementary approaches to identify cellular factors required for ANDV entry into human lung microvascular endothelial cells. We screened an siRNA library targeting 140 genes involved in membrane trafficking, and identified 55 genes required for ANDV infection. These genes control the major endocytic pathways, endosomal transport, cell signaling, and cytoskeleton rearrangement. We then used infectious ANDV and retroviral pseudovirions to further characterize the possible involvement of 9 of these genes in the early steps of ANDV entry. In addition, we used markers of cellular endocytosis along with chemical inhibitors of known endocytic pathways to show that ANDV uses multiple routes of entry to infect target cells. These entry mechanisms are mainly clathrin-, dynamin-, and cholesterol-dependent, but can also occur via a clathrin-independent manner.

## Introduction

Andes virus (ANDV) is a major representative of the New World hantaviruses in the Americas. It causes hantavirus pulmonary syndrome (HPS) with case fatality rates as high as 40% [1, 2]. HPS is characterized by fever, muscle aches, and headaches, rapidly progressing to pulmonary edema due to microvascular leakage, and to respiratory failure or shock [3]. At present, ANDV is the only hantavirus shown to be capable of human-to-human transmission [4]. No effective vaccines or antiviral drugs exist for HPS.

Hantaviruses are divided into Old World hantaviruses, such as Hantaan virus (HTNV) and Puumala virus (PUUV), and New World hantaviruses like ANDV and Sin Nombre virus. Hantaviruses belong to the *Bunyaviridae* family, and have a tri-segmented, negative-sense, single-stranded RNA genome. The genome consists of S, M, and L segments encoding the nucleocapsid (N) protein, 2 glycoproteins (Gn and Gc) produced from a single precursor (GPC), and L protein (also known as RNA-dependent RNA polymerase, RdRp), respectively. Attachment of ANDV to its receptor on host cells is mediated by the virus surface glycoproteins Gn and Gc. Virion uptake by the infected cells is followed by low pH-dependent fusion between the virus and the endosomal membranes, and the release of ribonucleocapsid cores into the cytoplasm [5].

Hantaviruses use integrins to enter host cells, and pathogenic hantaviruses like ANDV depend on integrin β3 (ITB3) as their receptor [6–8]. Cell susceptibility to hantavirus, however, depends not only on the expression of ITB3 [9, 10]; other cellular factors, such as decay-accelerating factor (DAF1) and the receptor of complement C1q are also important in hantavirus entry [11–14]. In addition, integrin β2 was recently identified as a receptor for HTNV and as responsible for hantavirus pathogenesis [15]. The precise roles of each of these factors in hantavirus cell entry are currently unclear, however.

After attaching to the cell membrane, viruses commonly use host endocytic pathways, such as clathrin-mediated endocytosis, caveolin-mediated endocytosis, and macropinocytosis to reach intracellular compartments. The major endocytic pathways can be distinguished on the basis of their differential sensitivity to chemical inhibitors [16]. Using such compounds, previous studies have shown that HTNV entry involves clathrin and dynamin, but not caveolin-mediated endocytosis [17, 18]. However, ANDV infection was shown to be independent of both clathrin- and caveolin-mediated endocytosis, suggesting that New and Old World hantaviruses differentially utilize host cytoskeletal components during their life cycles [18]. Recent reports have shown consistently that both HTNV and ANDV require cholesterol for cell entry, an indication of raft-dependent access [12, 19, 20].

Vascular endothelial cells are the primary targets of ANDV infection in humans [21], and infection in these cells leads to loss of capillary integrity [22]. To better simulate natural ANDV infection, we used primary human lung microvascular endothelial cells (HMVEC-L) to study ANDV cell entry. Identifying cellular factors required for ANDV entry into its human target cells is crucial for understanding how this virus functions, and the results would benefit future development of antiviral treatments for ANDV infection. To identify such factors, we conducted an siRNA screen that specifically targeted human genes required for endocytosis, intracellular vesicular transport, cell signaling, and cytoskeleton rearrangement. In addition, we used a panel of chemical inhibitors of endocytic pathways to further probe the mechanisms of ANDV entry.

## Materials and Methods

### Cell lines, virus, and antibodies

HMVEC-L (Lonza, Walkersville, MD, USA) were grown with EGM-2MV medium (Lonza) in cell culture flasks pre-coated with phosphate-buffered saline (PBS) containing 0.2% gelatin (Sigma-Aldrich, St. Louis, MO, USA). ANDV (strain Chile 9717869) was propagated in Vero-E6 cells (obtained from ATCC, Manassas, VA, USA) in a biosafety level 3 laboratory. Viral titers were determined using immunostaining as described previously [23]. Western blot analyses were used to validate siRNA knockdown of targeted genes, and were performed as described [23]. The antibodies used for western blotting were directed against ANDV Gc (1:2000 dilution; catalog no. H1808-60, US Biological, Swampscott, MA, USA), AP2M1 (1:1000; ab75995, Abcam, Cambridge, MA, USA), ARF6 (1:200; sc-7971, Santa Cruz Biotechnology, Santa Cruz, CA, USA), caveolin-1 (CAV1; 1:1000; 610058, BD Biosciences, San Jose, CA, USA), CDC42 (1:1000; PA1-092, Thermo Fisher Scientific, Rockford, IL, USA), clathrin heavy chain (CLTC; 1:500; MA1-065, Thermo Fisher Scientific), dynamin-2 (DNM2; 1:2000; sc-166669, Santa Cruz), ITB3 (1:100; sc-365670, Santa Cruz), NSF (1:2000; ab16681, Abcam), RAB5C (1:500; SAB4502559, Sigma-Aldrich), TSG101 (1:200; sc-7964, Santa Cruz), and β-actin (Sigma-Aldrich).

### Screening the siRNA library

The siRNA library used here (ON-TargetPlus, catalog no. G-105500, Dharmacon, Lafayette, CO, USA) targets human endocytic and membrane trafficking-related genes. A total of 140 genes was targeted using 4 siRNAs pooled for each gene [24]. All transfections were performed in a 96-well plate format. A 0.5% (v/v) stock of transfection reagent (DharmaFECT 1) was prepared in Opti-MEM solution (Life Technologies) and incubated at 23°C for 5 min. An equal volume (10 μL) of transfection reagent stock was mixed with rehydrated siRNA in each well for 20 min at 23°C. Subsequently, 2 × 10^4^ HMVEC-L in 80 μL of EGM-2MV were added to each well and incubated for 48 h to allow significant gene knockdown by siRNA. The final concentration of pooled siRNA was 50 nM. Cell viability assays were also performed in a duplicate experiment to monitor for any cell toxicity caused by siRNA transfection.

For screening purposes, after transfection with siRNA, ANDV was added to HMVEC-L at an MOI of 0.5. At 48 h post infection, the cells were fixed at -20°C in absolute methanol (Sigma-Aldrich) for 20 min. The cells were then subjected to a previously described immunolabeling assay [23]. Briefly, the cells were washed 3 times with PBS containing 0.1% v/v Tween-20 (PBS-T), blocked with 5% skim milk in PBS-T, and incubated at 37°C for 30 min. Plates were then washed with PBS-T, incubated with mouse monoclonal anti-PUUV N protein antibody (1:4000) for 30 min at 37°C, and washed as above. Plates were incubated with 1% H_2_O_2_ (Sigma-Aldrich) for 15 min at room temperature, and washed as above. Subsequently, goat anti-mouse IgG1-conjugated HRP (1:25,000; Southern Biotech, Birmingham, AL, USA) was added at 37°C for 30 min before washing as above. Signal was detected with chemiluminescent peroxidase substrate-3 (Sigma-Aldrich) on a Synergy HT Microplate Reader (Biotek, Broadview, IL, USA) using 0.1 s integration.

In addition to transfection reagent (DharmaFECT 1) alone, negative controls used were a non-targeting siRNA, RISC-free siRNA, and siRNA targeting the housekeeping genes cyclophilin B, lamin A/C, and glyceraldehyde-3-phosphate dehydrogenase (GAPDH) (all from Dharmacon). As positive controls, we included siRNA specifically targeting ANDV genome segments S or L, which suppressed expression of ANDV N protein in HMVEC-L as described in [23]. The quality and reliability of the screening result was determined by Z factor (Z′), which describes the available signal window for an assay in terms of the total separation between negative and positive controls minus the error associated with each type of control [25]. It is defined as

$$Z\prime =1-3\times \frac{\sigma_{p}\;+{\;\sigma }_{n}}{\mu_{p}-\;\mu_{n}}$$

where σ_p_, σ_n_, μ_p_, and μ_n_ are the standard deviations (σ) and the averages (μ) of the positive (p) and negative (n) controls.

### ANDV pseudovirion entry assays

An expression vector encoding the human codon-optimized ANDV *GPC* gene was synthesized (DNA2.0, Menlo Park, CA, USA), and HIV pseudoparticles bearing ANDV GPC were prepared as described previously [26]. Briefly, 1 × 10^6^ 293T-LentiX cells (Clontech, Mountain View, CA, USA) were co-transfected with plasmid DNA encoding the HIV genome containing the firefly luciferase gene (pNL4-3.Luc.R^-^E^-^) and an expression vector encoding the vesicular stomatitis virus (VSV) G protein or ANDV GPC. The ratio of HIV plasmid to glycoprotein expression vector was 8:1. Pseudoparticles were harvested from the medium of 293T-LentiX cells 48 and 72 h post transfection, and supernatants were combined and filtered to remove cellular debris. Pseudoparticle titers were determined on HMVEC-L cells using the LentiX provirus quantitation kit (Clontech). To measure ANDV glycoprotein-dependent entry, pseudoparticles were added to HMVEC-L cells and allowed to adsorb for 6 h. After this time, the inoculum was removed and replaced with phenol red-free EGM-2-MV medium (Lonza). Two days after infection, firefly luciferase levels were measured using the luciferase assay system (Promega, Madison, WI, USA).

### Chemical inhibitor studies

HMVEC-L were incubated at 37°C in low-serum (1% FBS) EGM-2MV medium supplemented with the following inhibitors: dynasore (ab120192, Abcam), chlorpromazine (CPZ; C8138, Sigma-Aldrich), Pitstop 2 (ab120687, Abcam), methyl-β-cyclodextrin (MβCD; C4555, Sigma-Aldrich), 5-(N-ethyl-N-isopropyl) amiloride (EIPA; A3085, Sigma-Aldrich), LY294002 (S1105, Selleckchem), or cytochalasin D (C8273, Sigma-Aldrich). Cytotoxic effects of these inhibitors on HMVEC-L were determined using the CellTiter-Glo luminescent assay (Promega) according to the manufacturer’s instructions, and were expressed as cytotoxic concentrations for 20% of the cells (CC_20_). For inhibitor treatment prior to ANDV or pseudovirion infection (pre-treatment), cells were treated with inhibitor diluted in low-serum (1% FBS) EGM-2MV medium or with an equivalent amount of a DMSO vehicle control for 1 h, and ANDV (MOI = 0.5) or pseudoparticles (MOI = 1) was added to cells in medium containing fresh inhibitor. After 3 h (ANDV) or 6 h (pseudovirions), the inoculum was removed and replaced with fresh medium containing inhibitor. Two days after ANDV infection, the cells were fixed in methanol, washed, and stained with PUUV anti-N protein primary antibodies, followed by AlexaFluor 488-conjugated secondary antibodies (Life Technologies, Grand Island, NY, USA). Cell nuclei were counterstained with DAPI (1 μg/mL, Life Technologies), and the plates were then imaged on Operetta High Content Screening system (Perkin Elmer, Waltham, MA, USA) in wide-field fluorescence mode using the 20× long WD objective. Twenty fields per well were imaged and analyzed. Subsequent image analyses were performed on Harmony software (Perkin Elmer) based on counting DAPI-stained nuclei as total cell populations followed by texture analysis of AlexaFluor 488-stained cytoplasms to determine the number of virus-infected cells. In the case of pseudovirion infection, luciferase assays were performed as described above.

For inhibitor treatment after ANDV infection (post-treatment), virus was added to cells in media without inhibitor; inhibitor was added 3 h later. All subsequent procedures were performed as described above.

### Dextran and transferrin uptake assay

HMVEC-L were incubated in ice-cold, low-serum (1% FBS) EGM-2MV medium for 30 min before inoculation with ANDV (MOI = 30) for 1 h at 4°C. The cells were washed with ice-cold PBS, and given ice-cold, low-serum EGM-2MV media containing 10 kDa dextran (250 μg/mL) or transferrin (100 μg/mL) conjugated to AlexaFluor 488 (Life Technologies); the cells were then incubated for 20 min on ice. Cells were transferred to 37°C for 15 or 30 min, and then washed 3 times with ice-cold 2 mM PBS glycine (pH 2) before fixing cells with 4% paraformaldehyde in PBS, permeabilizing in 0.1% Triton X-100 in PBS, and imaging for immunofluorescence. Briefly, cells were stained with polyclonal anti-ANDV primary antibodies and AlexaFluor 633-conjugated secondary antibodies (Life Technologies), and cell nuclei were counterstained with DAPI (1 μg/mL). The resulting coverslips were examined by confocal microscopy.

## Results

### A human membrane trafficking siRNA library screen identified genes required for ANDV infection in HMVEC-L

To elucidate the pathways involved in the ANDV infectious cycle and identify the cellular genes essential for this process, we screened a commercially available library of siRNAs directed against genes involved in cellular transport. This screen was performed using primary HMVEC-L, a physiologically relevant cell type for ANDV replication. HMVEC-L were transfected with siRNAs, and infected with ANDV (MOI = 0.5) 48 h later. Forty-eight hours post infection, the cells were fixed and ANDV N protein was detected using a cell-based enzymatic assay [23]. A Z′ factor above 0.5 was consistently calculated, confirming the quality of the assay. Cell viability assays performed on mock-infected cells demonstrated that none of the siRNA pools tested reduced cell viability by more than 20% (data not shown).

**Fig 1:**
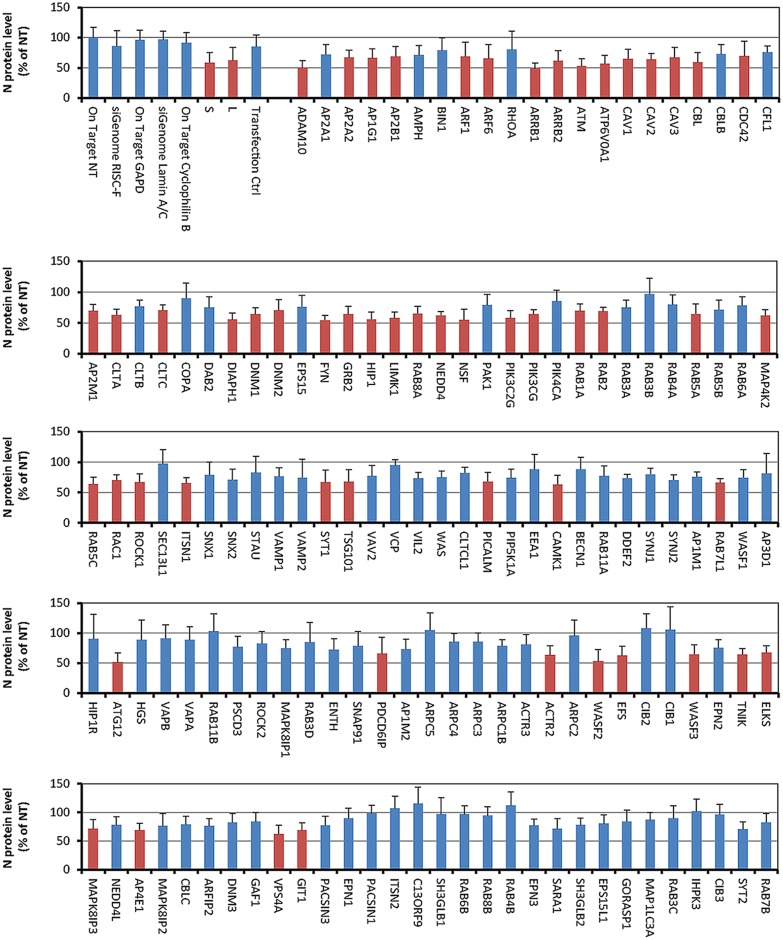
Using an siRNA library to identify genes involved in Andes virus infection of primary human lung cells. Andes virus (ANDV)-infected primary human macrovascular endothelial lung cells (HMVEC-L) after siRNA administration (blue) were compared with a negative control set and are presented as the percentage of ANDV-infected cells treated with non-targeting siRNA (NT). The results are shown as averages (± SD) of 4 independent sets of experiments. Negative control set includes non-targeting siRNA, RISC-free siRNA, and siRNAs targeting GAPDH, cyclophilin B, and lamin A/C. siRNA pools against the ANDV N segment were used to demonstrate positive knockdown. ANDV infection (shown in red) is significantly decreased after siRNA knockdown (p < 0.05).

Of the 140 siRNAs screened, 55 inhibited ANDV infection in HMVEC-L, as determined by analysis of variance (ANOVA) with Bonferroni correction (Fig 1 and S1 Table). These genes are known to regulate several endocytic pathways, vesicular transport, cell signaling, cytoskeletal formation, and exocytosis (S2 Table). None of the siRNAs tested increased ANDV replication. Of the 55 identified genes, 9 were selected for further characterization, based on their previously described association with entry pathways of various viruses (Table 1).

**Table 1:** Selected genes required for ANDV infection in HMVEC-L.

Gene	Gene/Protein Name	Function
***AP2M1***	Adaptor protein complex 2 subunit μ	AP-2 is involved in clathrin-dependent endocytosis in which cargo proteins are incorporated into vesicles surrounded by clathrin (clathrin-coated vesicles) destined for fusion with the early endosome. The AP-2 μ subunit binds to transmembrane cargo proteins; it recognizes Y-X-X-Phi motifs.
***ARF6***	ADP-ribosylation factor 6	A GTP-binding protein localized to the plasma membrane, ARF6 is involved in vesicular/protein trafficking, in regulating endocytic recycling and remodeling of membrane lipids, and in signaling pathways that lead to actin remodeling.
***CAV1***	Caveolin-1	Caveolin is the major component of the inner surface of caveolae, small invaginations of the plasma membrane. It is also a major scaffolding protein in caveolae-mediated endocytosis.
***CDC42***	Cell division control protein 42 homolog precursor	A plasma membrane-associated small GTPase which cycles between an active GTP-bound and an inactive GDP-bound state. Along with RAC1 and PAK1, it mediates signaling pathways to activate macropinocytosis.
***CLTC***	Clathrin heavy chain	Clathrin is a major protein component of the cytoplasmic face of intracellular organelles, called coated vesicles or coated pits. These specialized organelles are involved in intracellular trafficking of receptors and in endocytosis of a variety of macromolecules. The basic subunit of the clathrin coat is composed of 3 heavy chains and 3 light chains.
***DNM2***	Dynamin-2	Dynamin is a GTPase involved in producing microtubule bundles for vesicular trafficking processes, particularly endocytosis.
***NSF***	*N*-ethylmaleimide-sensitive factor	An ATPase required for vesicle-mediated transport, NSF is a component of the SNARE complex. It functions as a fusion protein required for the delivery of cargo proteins to endosomes and to all compartments of the Golgi stack independent of vesicle origin.
***RAB5C***	RAS-related protein RAB 5C	Members of the RAB protein family are small GTPases of the RAS superfamily, and are thought to ensure fidelity in docking and/or fusion of vesicles with their correct acceptor compartment.
***TSG101***	Tumor susceptibility gene 101 protein	A component of the ESCRTI complex, TSG101 binds to ubiquitinated cargo proteins and is required for sorting endocytic ubiquitinated cargos into multivesicular bodies. It also mediates the association between the ESCRT0 and ESCRTI complexes.

### Reducing expression of endocytic genes affects ANDV protein expression and virus release

**Fig 2:**
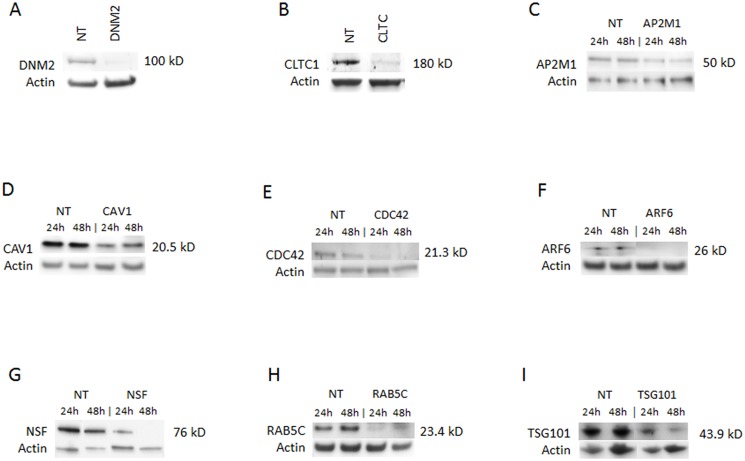
Knockdown of selected genes required for ANDV infection. Western blots of indicated proteins after knockdown with gene-specific siRNA against (A) dynamin 2 (DNM2); (B) clathrin heavy chain (CLTC); (C) AP2M1; (D) caveolin 1 (CAV1); (E) CDC42; (F) ARF6; (G) NSF; (H) RAB5C; or (I) TSG101; or non-targeting siRNA transfection control (NT). siRNAs were transfected into HMVEC-L at the concentration of 100 nM for 48 h (see Table 1 for more information regarding these genes). The cells were then infected with ANDV (MOI = 0.5) for 24 h or 48 h. Western blots were performed post infection to ensure knockdown of the specific protein expression. Molecular weight of each specific protein is indicated on the right side of each panel. The blots were also probed with β-actin specific antibody as the gel-loading control. (A) and (B) show only the results collected at 48 h.

**Fig 3:**
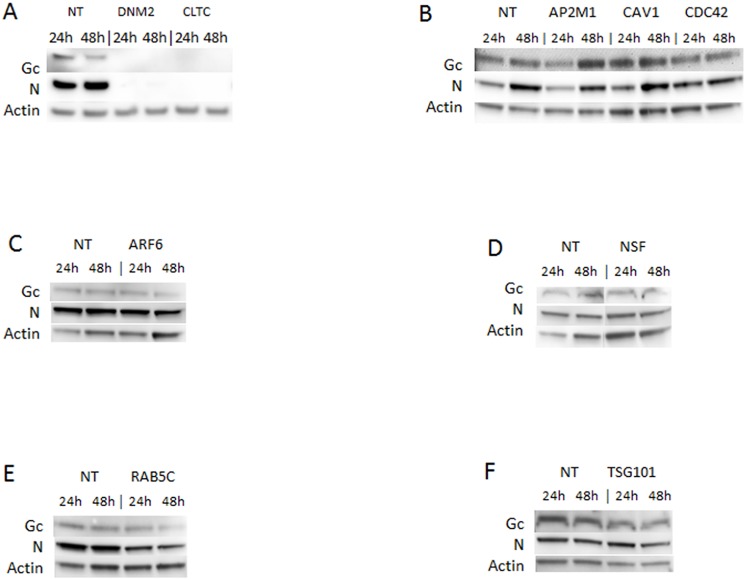
Effects of knocking down selected genes on ANDV replication. Western blots showing ANDV glycoprotein (Gc) and nucleoprotein (N) in HMVEC-L transfected with gene-specific siRNAs against (A) DNM2 or CLTC; (B) AP2M1, CAV1, or CDC42; (C) ARF6; (D) NSF; (E) RAB5C; or (F) TSG101; or with the non-targeting siRNA transfection control (NT). siRNAs were transfected into HMVEC-L at the concentration of 100 nM for 48 h. Cells were then infected with ANDV (MOI = 0.5) for 24 h or 48 h. Western blots were performed post infection to examine viral Gc and N protein expression. β-actin was probed as the loading control.

**Fig 4:**
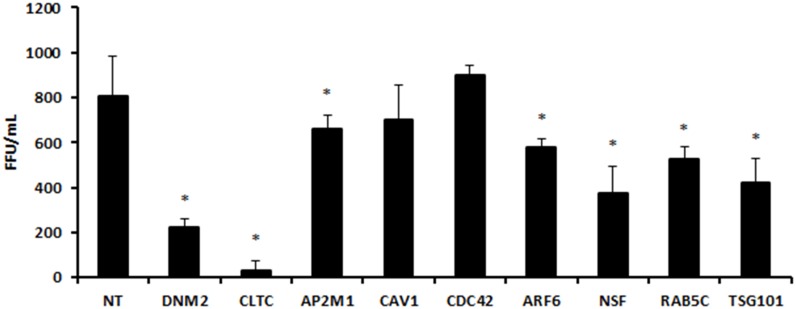
Knocking down selected host proteins by siRNA decreases ANDV release. Plaque assays to determine titers of ANDV released by HMVEC-L infected with ANDV after transfection with gene-specific siRNA against DNM2, CLTC, AP2M1, CAV1, CDC42, ARF6, RAB5C, NSF, or TSG101, or with the non-targeting siRNA transfection control (NT). siRNAs were transfected into HMVEC-L at the concentration of 100 nM for 48 h, and cells were infected with ANDV (MOI = 0.5) for 48 h. Culture media were harvested, and plaque assays were performed in Vero-E6 cells. Viral plaques were stained and counted 5 days post-infection and expressed in focus forming unit (FFU)/mL, as shown on the y-axis of each panel. Results presented are the averages (± SD) of 4 experiments. Statistically significant differences compared to NT results at the same time points are shown with an asterisk (*; p < 0.05).

We first used western blotting to assess the efficiency of knockdown mediated by each of the transfected siRNA pools. We found that siRNAs differed in knockdown efficiency, ranging from 57% to 100% (densitometry data not shown, and Fig 2). The effect of knocking down each cellular gene on ANDV replication was assessed by western blotting for the viral proteins N and Gc (Fig 3), and by determining the titers of virus released from the transfected and infected cells (Fig 4).

Several siRNA pools induced a dramatic reduction in ANDV protein expression and titers. These included siRNAs targeting DNM2 and CLTC, which reduced ANDV protein expression to undetectable levels (Figs 2A, 2B and 3A). Furthermore, siRNAs targeting CLTC reduced virus release from infected cells to nearly undetectable levels (Fig 4).

The knockdown of CAV1, a major scaffolding protein involved in caveolin-mediated endocytosis [27], was only partial (Fig 2D), so the role of caveolin in ANDV entry was unclear. Suppressing the small GTPase ARF6 (Fig 2F), which is involved in many aspects of vesicular trafficking and actin remodeling and is implicated in macropinocytosis [28, 29], did not significantly affect virus protein expression and titers (Figs 3C and 4). Similarly, knocking down the small GTP-binding protein CDC42 (Fig 2E), an upstream actin regulator involved in the formation of filopodia in macropinocytosis [30, 31], minimally reduced ANDV protein expression and titers (Figs 3B and 4). The apparent differences between the initial library screening and the results obtained in these experiments may reflect differences between the assays used, as well as variability in the extent of knockdown of the targeted genes.

Interestingly, knocking down AP2M1 (Fig 2C), a component of an accessory complex commonly associated with forming clathrin-coated pits on the plasma membrane [32], appeared to increase Gc and N protein levels in infected cells (Fig 3B). No reduction in N and Gc protein levels was detected after knocking down TSG101 (Fig 3F), but viral titers were reduced, suggesting that TSG101 does not affect entry or replication, but rather the subsequent assembly and release of progeny virions (Fig 4). Overall, our data indicate that ANDV entry and replication in HMVEC-L is clathrin- and dynamin-dependent.

### ANDV pseudovirion entry in HMVEC-L cells

**Fig 5:**
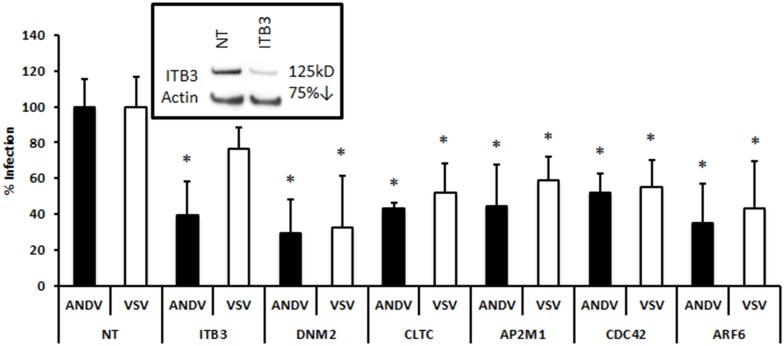
siRNA profiling of selected genes required for recombinant ANDV pseudovirion infection. Gene-specific siRNA against integrin β3 (ITB3), DNM2, CLTC, AP2M1, CDC42, or ARF6, or the non-targeting siRNA transfection control (NT), were transfected into HMVEC-L at the concentration of 100 nM for 48 h. Cells were infected with pseudovirions (MOI = 1) for 48 h. All pseudovirions consisted of an HIV backbone with firefly luciferase, and were pseudotyped with ANDV GPC (ANDV) or vesicular stomatitis virus G protein (VSV). Effects of gene knockdown were measured by quantifying luciferase activity in the host genome. Solid bars represent ANDV, and open bars represent VSV. Luciferase activity is expressed as units relative to non-targeting siRNA control (% infection). The effect of ITB3-specific siRNA knockdown is shown in the inset. Results presented are the averages (± SD) of 10 experiments (n = 10), and significant decreases in pseudovirion infection (p < 0.05) after siRNA knockdown are shown with an asterisk (*).

To determine if the identified genes played a role in ANDV entry or in other steps of ANDV replication, we used HIV pseudotyped particles bearing the ANDV glycoprotein. We have shown previously that entry and consequent expression of the luciferase reporter gene from these particles are ANDV glycoprotein-dependent [33]. HIV pseudovirions bearing the VSV glycoprotein G were used as a control. HMVEC-L cells were transfected with the specific siRNAs described above, and then transduced with pseudovirions bearing ANDV GPC or VSV G; luciferase levels were measured 2 days after infection (Fig 5).

As expected, knocking down ITB3 (Fig 5 inset), a known receptor for pathogenic hantaviruses like ANDV [6, 7], inhibited luciferase expression after infection with ANDV pseudovirions but not with VSV pseudovirions (Fig 5). This confirms the utility of the pseudovirus system for studying ANDV glycoprotein-dependent entry. Knocking down DNM2, CLTC, and AP2M1, factors involved in clathrin-mediated endocytosis, reduced luciferase expression (Fig 5), consistent with a role for this pathway in ANDV glycoprotein-dependent entry. Interestingly, knocking down CDC42 or ARF6 suppressed luciferase expression after infection with ANDV pseudovirions (Fig 5), though not with wild-type ANDV virus (Fig 3).

### Chemical inhibition suggests that ANDV uses clathrin-dependent cell entry in HMVEC-L

Next, we used a panel of chemical inhibitors of endocytosis to probe the endocytic pathways used by ANDV to enter primary HMVEC-L (Table 2). First, we tested the cytotoxic effects of each inhibitor in HMVEC-L cells, and subsequently used concentrations of inhibitors that minimally impacted cell viability (< 20% reduction). In general, these concentrations fell below those used in a previously published study on continuous cell lines [16].

**Table 2:** Chemical inhibitors of endocytic pathways used in this study.

Inhibitor	Endocytosis Pathway Blocked	Specific Activity	Toxicity to HMVEC-L (CC_20_)
Dynasore	Clathrin and caveolin	Blocks dynamins	87 μM
Chlorpromazine(CPZ)	Clathrin	Prevents coated pit formation by blocking AP2 binding to receptor	22 μM
Pitstop 2	Clathrin	Prevents coated pit formation by blocking attachment to clathrin heavy chain	16 μM
Methyl-β-cyclodextrin (MβCD)	Caveolin, clathrin, and macropinocytosis	Extracts cholesterol from cells	2 mM
5-(*N*-ethyl-*N*-isopropyl) amiloride (EIPA)	Macropinocytosis	Inhibits Na^+^/H^+^ exchange	51 μM
LY294002	Macropinocytosis	Inhibits PI3K α/β/δ	13 μM
Cytochalasin D	Clathrin and macropinocytosis	Disrupts actin polymerization	300 nM

**Fig 6:**
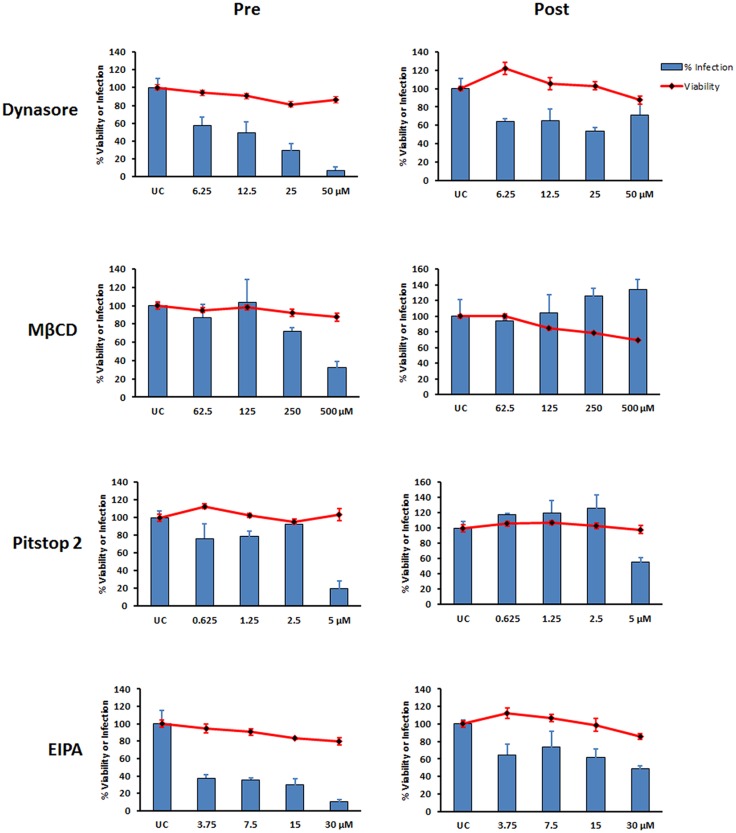
Productive ANDV infection in HMVEC-L depends on dynamin, cholesterol, and clathrin pathways. HUMVEC-L viability (red lines) and level of ANDV infection (blue bars) when using varying concentrations of the indicated inhibitors. For inhibitor pretreatment (Pre), HMVEC-L were treated with dynasore, MβCD, Pitstop 2, or EIPA for 1 h before inoculating with ANDV (MOI = 1) in the presence of the inhibitor. For post-treatment (Post), cells were infected with ANDV; after 3 h, virus was removed and media replaced with low-serum medium containing inhibitor. Infection was assessed 16 h post infection by immunofluorescence assay. Percentages of infected and viable cells were determined relative to untreated controls (UC). Results presented are the averages (± SD) of triplicate experiments. Recombinant VSV virus (rVSV) infection was also used as a control (S3 Fig).

Each chemical inhibitor was added either 1 h before or 3 h after virus infection, and was present during the course of the infection. Among the compounds that were found to inhibit ANDV infection was the dynamin inhibitor dynasore, which is required for the scission step that separates the vesicle from the plasma membrane (in clathrin- or caveolin-mediated endocytosis) or from the intracellular organelle (Table 2). Pitstop 2, which blocks clathrin-mediated endocytosis (Table 2, [34]), also significantly reduced ANDV infectivity (Fig 6). Pitstop 2 is known to specifically work through blocking protein binding to the N terminus of clathrin, although it can affect other nonspecific targets in the cell [35]. MβCD, which depletes cellular cholesterol (Table 2) and inhibits ANDV infection in Vero-E6 cells [19], also blocked ANDV infection of HMVEC-L. EIPA is a potent and specific inhibitor of the Na^+^/H^+^ exchange, and is frequently used as a specific inhibitor of macropinocytosis [36]. When added prior to infection, EIPA potently inhibited ANDV infection of HMVEC-L in a concentration-dependent fashion (Fig 6).

In general, adding the compounds after ANDV infection had less effect on infectivity, suggesting that these compounds affect virus entry. Pretreating HMVEC-L cells with CPZ, a clathrin-mediated endocytosis inhibitor, and with PI3K inhibitor LY294002 did not inhibit virus entry (S1 Fig). Similarly, pretreating with cytochalasin D, a known inhibitor of actin polymerization, had no impact on ANDV infectivity in HMVEC-L (S1 Fig). However, HMVEC-L cells were extremely sensitive to cytochalasin D treatment, and even very low concentrations caused extensive cytopathic effect (S2 Fig).

**Fig 7:**
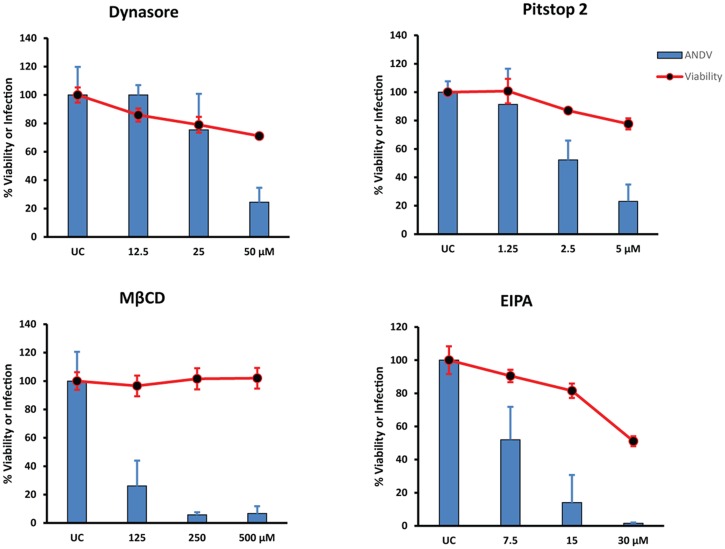
ANDV pseudovirion entry into HMVEC-L utilizes clathrin-mediated endocytosis and is cholesterol-dependent. Percent HMVEC-L viability (red lines) and ANDV infection (blue bars) after treatment with indicated inhibitors and infection with pseudovirions expressing luciferase and ANDV glycoprotein (GPC). HMVEC-L were treated with dynasore, MβCD, Pitstop 2, or EIPA as in Fig 6 before inoculating with ANDV pseudovirions (MOI = 1) in the presence of inhibitor. After 6 h, virus was removed and replaced with low-serum medium containing inhibitor. Infection was assayed 40 h later by measuring luciferase activity. Percentages of viable and infected cells were determined relative to untreated control (UC). Results presented are the averages (± SD) of triplicate experiments.

To confirm that the effects of EIPA, dynasore, Pitstop 2, and MβCD were specific for ANDV glycoprotein-dependent entry, we tested the ability of these inhibitors to block the entry of pseudovirions bearing ANDV GPC (Fig 7). Pretreating cells with each compound caused a concentration-dependent reduction in infection (Fig 7), confirming that these compounds inhibit ANDV entry into HMVEC-L. Interestingly, some of the compounds were more cytotoxic during these experiments than in ANDV-infected cells.

Altogether, these data suggest that ANDV uses clathrin- and cholesterol-dependent cell entry pathways, while inhibition with EIPA also implicates entry via a mechanism similar to macropinocytosis in HMVEC-L cells.

### ANDV partially co-localizes with both transferrin and fluorescent dextran molecules

**Fig 8:**
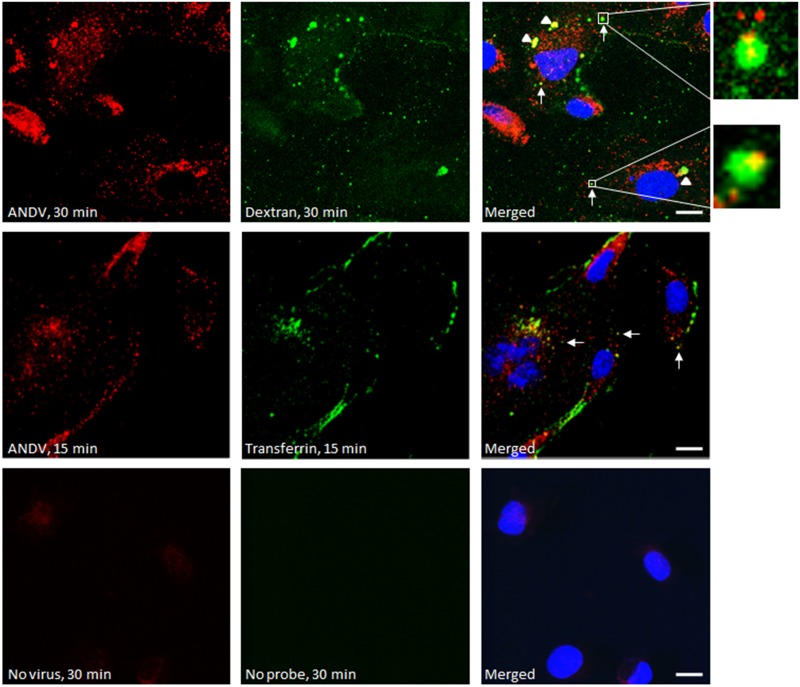
Co-localization of ANDV with high molecular weight dextran and transferrin in HMVEC-L. Confocal images of HMVEC-L treated with high molecular weight dextran or transferrin and infected with ANDV. ANDV is shown in red and was stained with anti-ANDV polyclonal antibodies and AlexaFluor 633-conjugated secondary antibodies. Green represents 10 kDa dextran or transferrin conjugated to AlexaFluor 488. Host nuclei were stained blue with DAPI. Confocal images were taken 15 or 30 min post infection, as indicated. White arrows indicate co-localization of ANDV with dextran or transferrin. Arrowheads show areas of intracellular viral accumulation. Bar, 10 μm.

To further elucidate any possible role of macropinocytosis in ANDV entry into primary lung cells, we traced the internalization of ANDV and dextran, a fluorescent fluid phase marker [37]. HMVEC-L were inoculated with ANDV (MOI = 30), washed, and incubated with dextran conjugated to AlexaFluor 488 (Fig 8, top row) or with labeled transferrin (Fig 8, middle row). Confocal microscopy revealed that ANDV virions (shown in red) colocalized with dextran (green, Fig 8, top row). At 30 min post virus entry, large inclusions were observed when cells were co-stained for both virus antigen and dextran. We found that the ratio of those large inclusions to the punctate staining also observed intracellularly was approximately 1:1. The size of the larger inclusions was variable, but they were significantly bigger than the punctate staining. We do not know if the large inclusions are physiologically relevant at this point. The fact that they are not present at 15 min post entry (second row in the Fig 8) indicates that they are not virus aggregates created during virus stock preparation, but are areas of intracellular virus accumulation. These results suggest that ANDV can enter primary cells via multiple endocytotic mechanisms, including clathrin-dependent endocytosis, and possibly implicate macropinocytosis in virus entry.

## Discussion

In the present study, we identified cellular pathways by which ANDV enters primary HMVEC-L, a physiologically relevant cell type. We initially used an siRNA library to identify important genes, and subsequently used chemical inhibitors of endocytosis to probe ANDV cell entry mechanisms. Our data indicate that ANDV enters primary human lung cells by clathrin-mediated endocytosis, though some evidence for an additional macropinocytosis-like entry mechanism was also found.

Viruses can use several pathways to enter cells, although many of them will preferentially use one pathway, depending on the cell type [38–42]. Hantavirus infection is a multi-step process initiated by cell surface attachment and followed by internalization of virions, converging toward the early endosome [5]. A previous study showed that pre-treatment with chlorpromazine or filipin did not significantly decrease ANDV infection, and concluded that ANDV does not use either clathrin- or caveolin-mediated endocytosis to enter Vero-E6 cells [18]. Another study showed that ANDV entry is inhibited by the cholesterol inhibitor mevastatin in Vero-E6 cells, suggesting that entry may occur through endocytosis via lipid rafts [19]. In addition to these seemingly contradictory findings, ITB3, a receptor for pathogenic hantaviruses including ANDV, is internalized by both clathrin- and non-clathrin-dependent endocytosis [43, 44].

We found that cellular genes like DNM2 and CLTC were essential for ANDV entry into HMVEC-L, suggesting entry via clathrin-mediated endocytosis. These results were supported by inhibition of ANDV and pseudovirion infection by the chemical inhibitors dynasore and Pitstop 2.

CPZ is an antipsychotic agent that has been implicated in clathrin-mediated endocytosis because it blocks the interaction of AP2 with clathrin during the formation of clathrin-coated pits. However, CPZ can also bind to other proteins, including calmodulin, channel proteins, and other membrane proteins, and more recently has been shown to trigger autophagy without causing cell death [45]. Actually, treatment with CPZ did not inhibit ANDV infection, but rather enhanced ANDV infection (S1A Fig). A probable explanation for this result is that CPZ is known to induce cell autophagy, and ANDV utilizes autophagy for efficient replication [33, 46]. CPZ treatment is also known to inhibit virus entry in continuous cell lines, but not in a number of primary cell lines, particularly in HMVEC-L cells [38].

Unlike clathrin-dependent endocytosis, no single protein coat, cellular factor, chemical inhibitor, or feature defines macropinocytosis [16, 31]. Experimental criteria have been recommended for determining whether a virus uses the macropinocytic pathway, with the caveat that the extent of entry through this pathway can differ with cell type [31]. Na^+^/H^+^ exchangers, such as EIPA, are understood to be important indicators of viral entry by macropinocytosis, and we found that EIPA potently inhibited ANDV entry. Moreover, ANDV co-localized with high molecular weight dextran within intracellular vacuoles. Macropinocytosis can be a direct viral entry mechanism, or be used indirectly, by virions first binding to receptors in clathrin-coated pits, and then being enclosed by lamellipodia or circular ruffles to complete the engulfing process [31]. Whether such sequential, coordinated actions between endocytic pathways act during ANDV entry remains to be elucidated.

Actin is required for clathrin-mediated endocytosis or macropinocytosis [47]. As mentioned earlier, treatment with EIPA, which can also induce reorganization of the actin structure [48], suppressed ANDV entry by these mechanisms. However, the actin polymerization inhibitor cytochalasin D had no impact on ANDV infectivity in HMVEC-L at the maximum concentration used (250 nM). The reason for these seemingly contradictory results is that cytochalasin D caused extensive cytotoxicity to HMVEC-L cells before reaching the concentrations of 2–3 μM known to be necessary to disrupt actin polymerization [49]. In addition to cytochalasin D, pretreating HMVEC-L cells with PI3K inhibitor LY294002 did not inhibit virus entry, also indicating that ANDV entry is PI3K-independent. These results are shown in S1 Fig.

Interestingly, knocking down macropinocytosis markers PAK-1 (Fig 1), CDC42 (Figs 3 and 4), and ARF6 (Figs 3 and 4) did not significantly reduce ANDV infection. However, another main marker of macropinocytosis, Rho GTPase RAC1, significantly contributed to ANDV infection, as shown in Fig 1. It is possible that when Rho GTPases like CDC42 are inhibited, other GTPases substitute their function. Knocking down genes encoding WASF2 and WASF3, members of the Wiskott-Aldrich syndrome protein family, also affected virus replication (Fig 1). These gene products form a multiprotein complex that links receptor kinases and GTPases with actin and ultimately facilitates non-clathrin mediated endocytosis.

While validating the involvement of endocytic genes identified in the initial siRNA library screening, we found that knocking down NSF and TSG101 had little effect on the expression of ANDV N and Gc proteins, but significantly reduced virus release, suggesting that these proteins are involved in viral assembly or budding, but not in entry or replication. These proteins, or their associated proteins, are thus potential cellular targets for anti-ANDV therapies.

In conclusion, our results suggest that ANDV can use clathrin-mediated endocytosis to enter a physiologically relevant host cell type. ANDV entry is also dynamin- and cholesterol-dependent. Additionally, a clathrin-independent pathway is involved; this pathway shares some features with macropinocytosis, but the exact mechanism remains to be determined. These pathways should be considered when designing antiviral therapies targeting the entry mechanism.

## Supporting Information

S1 Fig Effects of chlorpromazine, cytochalasin D, and LY294002 on ANDV infection in HMVEC-L. Viability curve (red line) and percentage of ANDV-infected HMVEC-L (blue bars) after treatment with indicated concentrations of (A) chlorpromazine, (B) cytochalasin D, or (C) LY294002. Cells were incubated with inhibitor for 1 h prior to ANDV infection (MOI = 1). After 3 h, virus was removed and replaced with low-serum medium containing inhibitor. ANDV presence was determined 16 h after infection by immunostaining the N protein. Viability and percentages of infected cells were calculated relative to untreated controls (UC). Results presented are the averages (± SD) of triplicate experiments.
(TIF)

S2 Fig Cytotoxicity of cytochalasin D in HMVEC-L. Viability curve of HMVEC-L after treatment with indicated concentrations of cytochalasin D. Cells were incubated with inhibitor for 16 h before viability was determined relative to untreated controls (UC). Results presented are the averages (± SD) of triplicate experiments.
(TIF)

S3 Fig Effects of Pitstop 2, EIPA, MβCD, dynasore, and chlorpromazine on recombinant VSV infection in HMVEC-L. Viability curve (red line) and percentage of rVSV-infected HMVEC-L (blue bars) after treatment with indicated concentrations of (A) Pitstop 2, (B) EIPA, (C) MβCD, (D) dynasore, or (E) chlorpromazine. Cells were incubated with inhibitor for 1 h prior to rVSV infection (MOI = 1). After 3 h, virus was removed and replaced with low-serum medium containing inhibitor. rVSV presence was determined 8 h after infection by the fluorescent intensity of GFP expressed from rVSV. rVSV was produced from Vero-E6 cells transfected with KeraFAST VSV-ΔG-GFP plasmid expression vectors and pseudotyped with VSV G. Viability and percentages of infected cells were calculated relative to untreated controls (UC). Results presented are the averages (± SD) of triplicate experiments.
(TIF)

S1 Table Results of siRNA screening. (DOCX)

S2 Table Genes identified by siRNA library screening that affect ANDV infection of HMVEC-L. (DOC)

S3 Table Viral protein levels and ANDV release determined by western blotting and plaque assays, respectively. (DOCX)
