# Supplementary figures and images for: Endocytic Pathways Used by Andes Virus to Enter Primary Human Lung Endothelial Cells
Source: PLoS One. 2016 Oct 25;11(10):e0164768. doi: 10.1371/journal.pone.0164768 (PMC5079659; doi:10.1371/journal.pone.0164768)

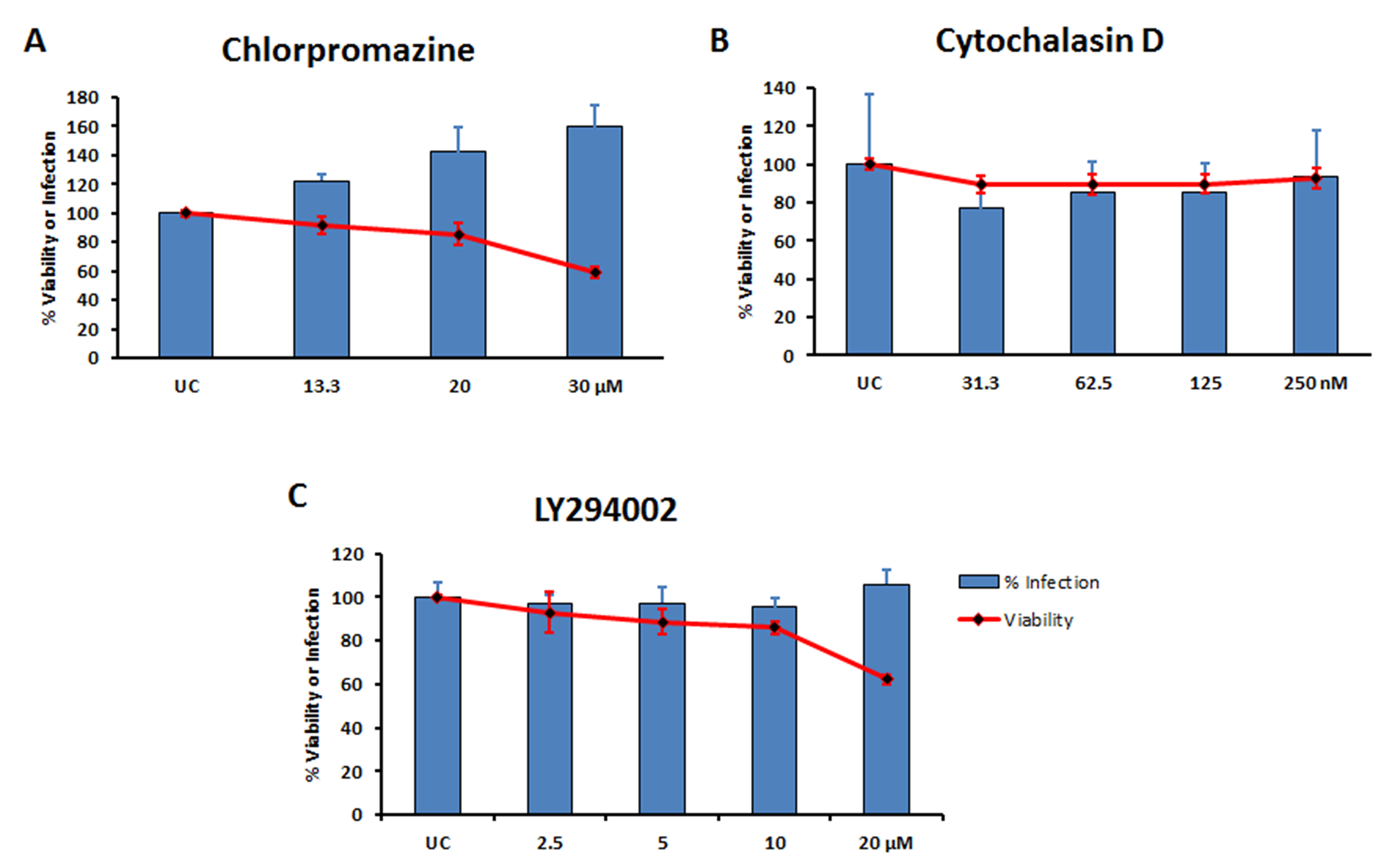

Supplement: S1 Fig — Viability curve (red line) and percentage of ANDV-infected HMVEC-L (blue bars) after treatment with indicated concentrations of (A) chlorpromazine, (B) cytochalasin D, or (C) LY294002. Cells were incubated with inhibitor for 1 h prior to ANDV infection (MOI = 1). After 3 h, virus was removed and replaced with low-serum medium containing inhibitor. ANDV presence was determined 16 h after infection by immunostaining the N protein. Viability and percentages of infected cells were calculated relative to untreated controls (UC). Results presented are the averages (± SD) of triplicate experiments. (TIF) [file pone.0164768.s001.tif]

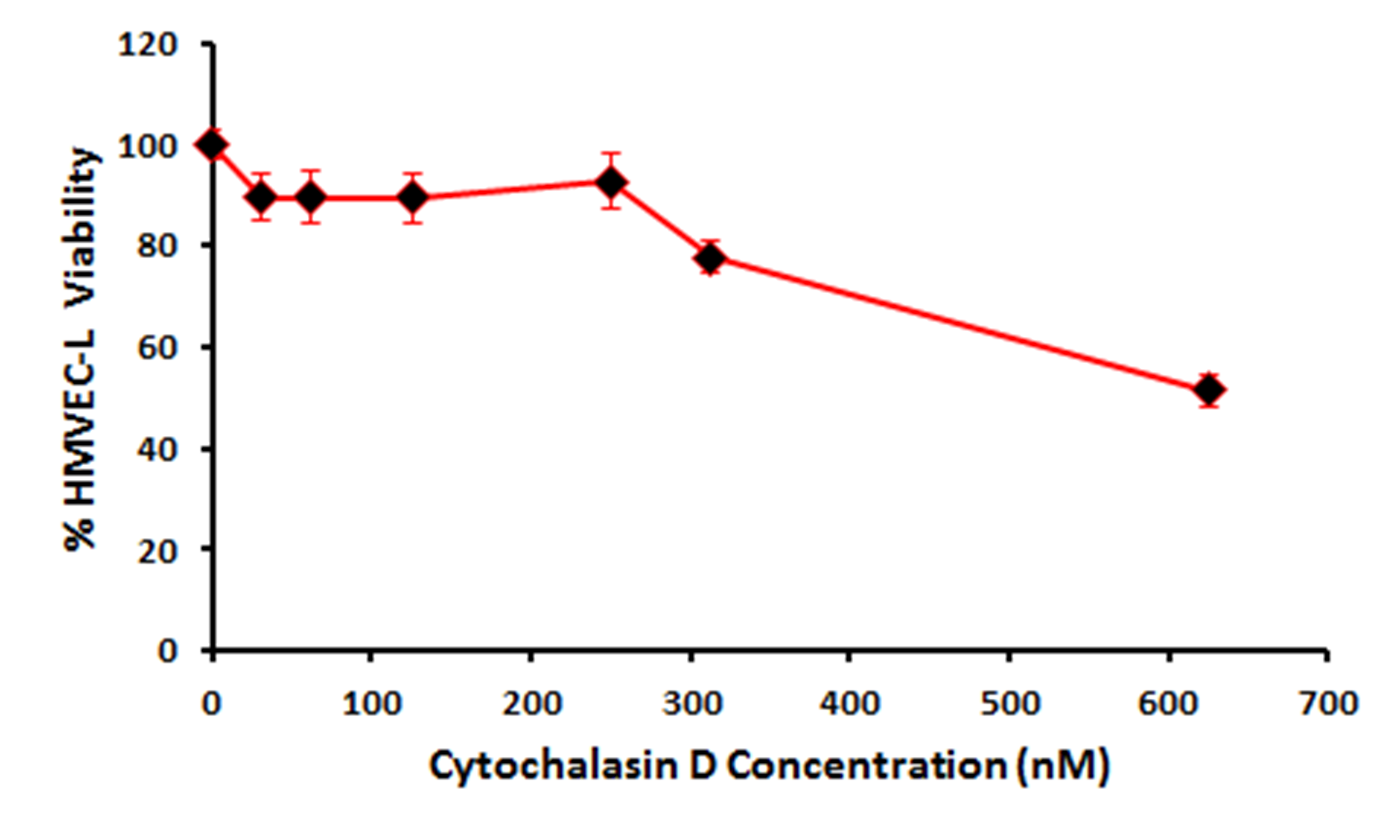

Supplement: S2 Fig — Viability curve of HMVEC-L after treatment with indicated concentrations of cytochalasin D. Cells were incubated with inhibitor for 16 h before viability was determined relative to untreated controls (UC). Results presented are the averages (± SD) of triplicate experiments. (TIF) [file pone.0164768.s002.tif]

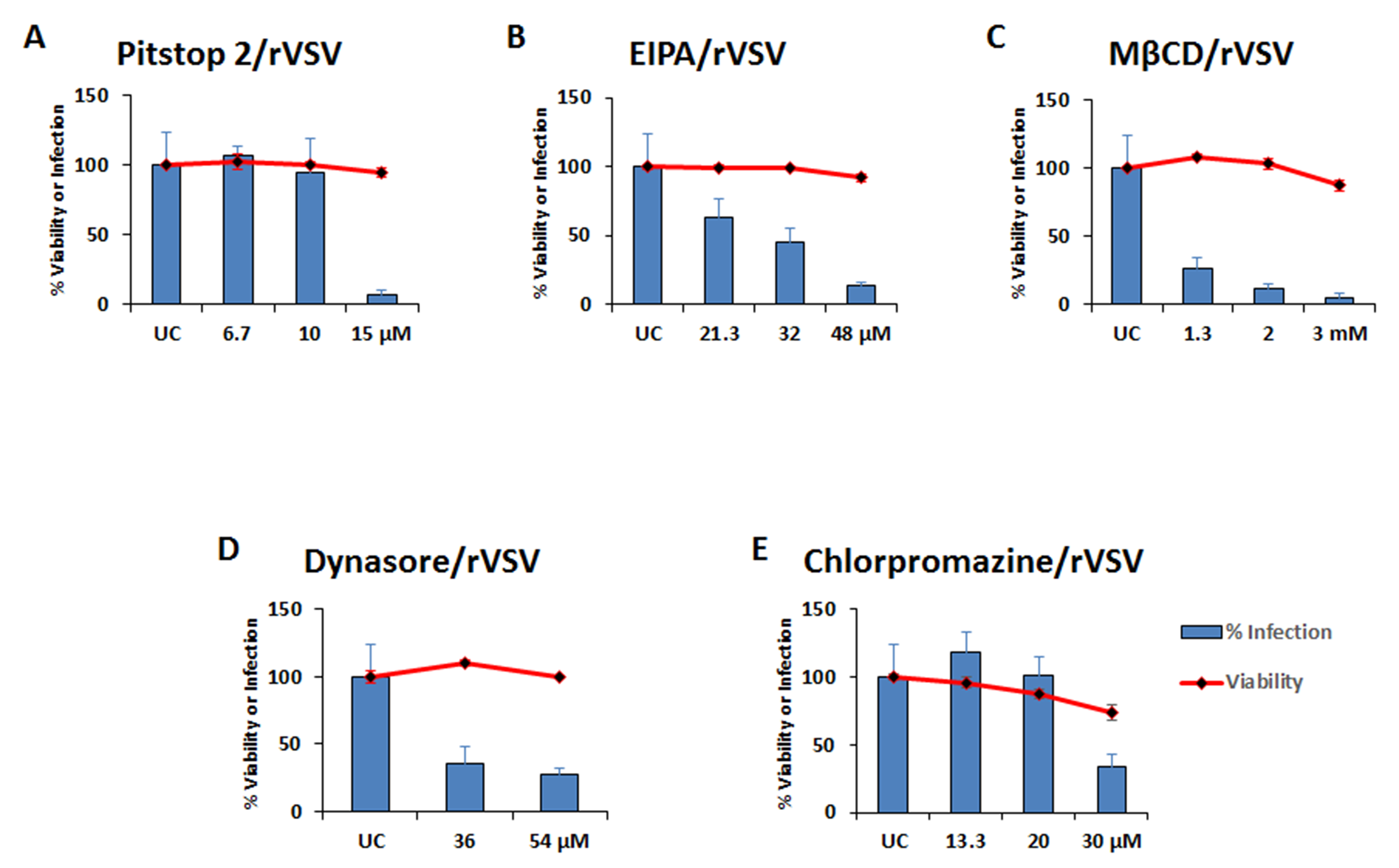

Supplement: S3 Fig — Viability curve (red line) and percentage of rVSV-infected HMVEC-L (blue bars) after treatment with indicated concentrations of (A) Pitstop 2, (B) EIPA, (C) MβCD, (D) dynasore, or (E) chlorpromazine. Cells were incubated with inhibitor for 1 h prior to rVSV infection (MOI = 1). After 3 h, virus was removed and replaced with low-serum medium containing inhibitor. rVSV presence was determined 8 h after infection by the fluorescent intensity of GFP expressed from rVSV. rVSV was produced from Vero-E6 cells transfected with KeraFAST VSV-ΔG-GFP plasmid expression vectors and pseudotyped with VSV G. Viability and percentages of infected cells were calculated relative to untreated controls (UC). Results presented are the averages (± SD) of triplicate experiments. (TIF) [file pone.0164768.s003.tif]
