# Supplementary material for: Endocytic Pathways Used by Andes Virus to Enter Primary Human Lung Endothelial Cells
Source: PLoS One. 2016 Oct 25;11(10):e0164768. doi: 10.1371/journal.pone.0164768 (PMC5079659; doi:10.1371/journal.pone.0164768)
Supplement: S1 Table — (DOCX) [file pone.0164768.s004.docx]

**S1 Table. Results of siRNA screening.**

| **Control or Targeted Gene Symbol** | **Relative N Protein Level %^a^** | **Standard Deviation** |
| --- | --- | --- |
| On-Target NT | 100.00 | 17.01 |
| siGenome RISC-F | 86.29 | 25.02 |
| On-Target GAPD | 96.09 | 16.18 |
| siGenome Lamin A/C | 96.69 | 14.05 |
| On-Target Cyclophilin B | 91.33 | 17.02 |
| S | 58.28 | 17.02 |
| L | 62.58 | 21.00 |
| Transfection Control | 85.17 | 19.38 |
| ADAM10 | 50.49 | 11.06 |
| AP2A1 | 71.64 | 16.79 |
| AP2A2 | 67.00 | 12.28 |
| AP1G1 | 66.02 | 15.56 |
| AP2B1 | 68.60 | 17.07 |
| AMPH | 70.72 | 16.24 |
| BIN1 | 79.21 | 20.56 |
| ARF1 | 68.69 | 23.40 |
| ARF6 | 65.47 | 23.10 |
| RHOA | 80.28 | 30.29 |
| ARRB1 | 48.64 | 8.91 |
| ARRB2 | 61.39 | 17.01 |
| ATM | 52.82 | 12.21 |
| ATP6V0A1 | 56.83 | 13.38 |
| CAV1 | 64.43 | 16.01 |
| CAV2 | 63.48 | 10.15 |
| CAV3 | 67.11 | 16.80 |
| CBL | 59.10 | 15.92 |
| CBLB | 72.56 | 16.37 |
| CDC42 | 69.50 | 24.25 |
| CFL1 | 75.80 | 10.11 |
| AP2M1 | 69.78 | 10.26 |
| CLTA | 62.70 | 9.95 |
| CLTB | 76.96 | 10.38 |
| CLTC | 70.18 | 9.08 |
| COPA | 89.90 | 25.13 |
| DAB2 | 74.94 | 17.37 |
| DIAPH1 | 55.67 | 10.34 |
| DNM1 | 63.95 | 10.97 |
| DNM2 | 70.13 | 17.63 |
| EPS15 | 76.13 | 18.46 |
| FYN | 54.54 | 7.53 |
| GRB2 | 63.91 | 12.71 |
| HIP1 | 56.06 | 11.64 |
| LIMK1 | 57.74 | 10.24 |
| RAB8A | 65.29 | 11.88 |
| NEDD4 | 62.23 | 6.05 |
| NSF | 54.66 | 17.42 |
| PAK1 | 78.93 | 16.98 |
| PIK3C2G | 57.95 | 11.83 |
| PIK3CG | 64.16 | 6.98 |
| PIK4CA | 85.25 | 18.11 |
| RAB1A | 69.46 | 11.56 |
| RAB2 | 68.73 | 6.96 |
| RAB3A | 74.90 | 11.70 |
| RAB3B | 96.80 | 25.50 |
| RAB4A | 79.54 | 15.61 |
| RAB5A | 64.62 | 16.07 |
| RAB5B | 71.61 | 15.35 |
| RAB6A | 77.84 | 14.32 |
| MAP4K2 | 61.74 | 9.93 |
| RAB5C | 64.03 | 10.92 |
| RAC1 | 70.06 | 8.46 |
| ROCK1 | 66.64 | 13.63 |
| SEC13L1 | 97.34 | 22.94 |
| ITSN1 | 64.90 | 8.86 |
| SNX1 | 78.94 | 21.39 |
| SNX2 | 70.97 | 17.64 |
| STAU | 82.59 | 27.04 |
| VAMP1 | 76.17 | 14.57 |
| VAMP2 | 74.12 | 30.62 |
| SYT1 | 66.52 | 20.01 |
| TSG101 | 67.59 | 19.80 |
| VAV2 | 76.63 | 17.74 |
| VCP | 94.97 | 9.10 |
| VIL2 | 72.95 | 9.86 |
| WAS | 74.62 | 10.43 |
| CLTCL1 | 82.10 | 9.61 |
| PICALM | 67.94 | 14.63 |
| PIP5K1A | 73.84 | 14.76 |
| EEA1 | 87.76 | 25.04 |
| CAMK1 | 62.78 | 14.88 |
| BECN1 | 88.11 | 19.79 |
| RAB11A | 76.74 | 17.21 |
| DDEF2 | 73.43 | 6.55 |
| SYNJ1 | 79.62 | 10.31 |
| SYNJ2 | 70.34 | 8.56 |
| AP1M1 | 74.81 | 9.04 |
| RAB7L1 | 65.84 | 6.67 |
| WASF1 | 74.23 | 13.18 |
| AP3D1 | 80.66 | 33.46 |
| HIP1R | 89.87 | 40.89 |
| ATG12 | 51.08 | 15.25 |
| HGS | 88.35 | 33.28 |
| VAPB | 91.34 | 22.50 |
| VAPA | 88.41 | 21.60 |
| RAB11B | 102.77 | 28.62 |
| PSCD3 | 76.79 | 18.05 |
| ROCK2 | 82.44 | 19.76 |
| MAPK8IP1 | 74.61 | 14.07 |
| RAB3D | 84.33 | 32.97 |
| ENTH | 72.33 | 17.83 |
| SNAP91 | 78.23 | 24.19 |
| PDCD6IP | 65.54 | 27.36 |
| AP1M2 | 72.52 | 16.96 |
| ARPC5 | 104.39 | 28.64 |
| ARPC4 | 85.30 | 13.53 |
| ARPC3 | 85.74 | 14.04 |
| ARPC1B | 78.53 | 10.71 |
| ACTR3 | 80.35 | 17.47 |
| ACTR2 | 63.43 | 15.48 |
| ARPC2 | 95.70 | 25.76 |
| WASF2 | 52.69 | 19.84 |
| EFS | 62.75 | 14.90 |
| CIB2 | 107.84 | 24.11 |
| CIB1 | 105.55 | 37.98 |
| WASF3 | 64.21 | 15.90 |
| EPN2 | 75.10 | 14.06 |
| TNIK | 64.29 | 9.87 |
| ELKS | 67.10 | 11.23 |
| MAPK8IP3 | 71.16 | 16.14 |
| NEDD4L | 77.48 | 14.69 |
| AP4E1 | 68.39 | 12.18 |
| MAPK8IP2 | 75.69 | 22.93 |
| CBLC | 78.19 | 14.74 |
| ARFIP2 | 75.90 | 13.23 |
| DNM3 | 82.06 | 15.87 |
| GAF1 | 83.30 | 16.98 |
| VPS4A | 61.58 | 15.91 |
| GIT1 | 68.17 | 13.33 |
| PACSIN3 | 76.83 | 16.36 |
| EPN1 | 89.60 | 18.03 |
| PACSIN1 | 98.91 | 13.46 |
| ITSN2 | 106.85 | 20.96 |
| C13ORF9 | 114.93 | 29.14 |
| SH3GLB1 | 96.77 | 29.04 |
| RAB6B | 97.06 | 14.62 |
| RAB8B | 94.40 | 15.61 |
| RAB4B | 111.99 | 23.35 |
| EPN3 | 77.27 | 10.75 |
| SARA1 | 70.70 | 18.66 |
| SH3GLB2 | 77.73 | 12.42 |
| EPS15L1 | 80.53 | 14.95 |
| GORASP1 | 83.77 | 19.90 |
| MAP1LC3A | 86.51 | 13.01 |
| RAB3C | 89.21 | 22.54 |
| IHPK3 | 101.94 | 21.20 |
| CIB3 | 96.28 | 17.89 |
| SYT2 | 70.48 | 13.02 |
| RAB7B | 82.01 | 15.94 |

^a^ % values were determined relative to non-targeting siRNA control (On-Target NT).
