# Supplementary material for: Endocytic Pathways Used by Andes Virus to Enter Primary Human Lung Endothelial Cells
Source: PLoS One. 2016 Oct 25;11(10):e0164768. doi: 10.1371/journal.pone.0164768 (PMC5079659; doi:10.1371/journal.pone.0164768)
Supplement: S2 Table — (DOC) [file pone.0164768.s005.doc]

**S2 Table. Genes identified by siRNA library screening that affect ANDV infection of HMVEC-L.**

| **Gene Symbol** | **Gene Accession No.** | **Gene Name and Function** |
| --- | --- | --- |
| *ACTR2* | NM_005722 | ACTIN-RELATED PROTEIN 2; component of Arp2/3 complex that promotes actin assembly in lamellipodia and may participate in lamellipodial protrusion. |
| *ADAM10* | NM_001110 | A DISINTEGRIN AND METALLOPROTEINASE DOMAIN 10; cell surface protein with a unique structure possessing both potential adhesion and protease function. Possesses tumor necrosis factor-α (TNFα) convertase activity. |
| *AP1G1* | NM_001030007 | ADAPTOR-RELATED PROTEIN COMPLEX 1, γ-1 SUBUNIT; essential for the formation of adaptor complexes of clathrin-coated vesicles. |
| *AP2A2* | NM_012305 | ADAPTOR-RELATED PROTEIN COMPLEX 2, α-2 SUBUNIT; shares more than 97% sequence identity with rat α-adaptin. |
| *AP2B1* | NM_001282 | ADAPTOR-RELATED PROTEIN COMPLEX 2, β-1 SUBUNIT; β-adaptin subunit of the clathrin coat assembly complex. |
| *AP2M1* | NM_001025205 | ADAPTOR-RELATED PROTEIN COMPLEX 2, µ-1 SUBUNIT; component of the AP2 coat assembly protein complex of clathrin-coated vesicles. |
| *AP4E1* | NM_007347 | ADAPTOR-RELATED PROTEIN COMPLEX 4, ε-1 SUBUNIT; subunit of a novel type of clathrin- or non-clathrin-associated protein coat involved in targeting proteins from the trans-Golgi network to the endosomal-lysosomal system. |
| *ARF1* | NM_001658 | ADP-RIBOSYLATION FACTOR 1; involved in membrane trafficking; recruits and binds cytoplasmic COPI to the Golgi membranes. Constitutes a family of the RAS superfamily. |
| *ARF6* | NM_001663 | ADP-RIBOSYLATION FACTOR 6; part of a group of RAS-like small GTPases called ADP-ribosylation factors (ARFs) that are involved in intracellular membrane trafficking. |
| *ARRB1* | NM_020251 | ARRESTIN β1; inhibits the signaling function of BARK-phosphorylated β-adrenergic receptor β-arrestin 1 mutants that are impaired either in SRC binding or in the ability to target receptors to clathrin-coated pits. Acts as a dominant negative inhibitor of β2 adrenergic receptor-mediated activation of the MAP kinases ERK1 and ERK2. |
| *ARRB2* | NM_199004 | ARRESTIN β2; regulates receptor coupling to G proteins, and functions in internalization and signaling of these receptors. |
| *ATG12* | NM_004707 | AUTOPHAGY RELATED 12; ubiquitin-like protein involved in autophagy vesicle formation. Plays a role in translation or delivery of incoming viral RNA to the translation apparatus. |
| *ATM* | NM_138292 | ATAXIA-TELANGIECTASIA MUTATED GENE; member of the phosphatidylinositol-3 kinase family of proteins that respond to DNA damage by phosphorylating key substrates involved in DNA repair and/or cell cycle control. |
| *ATP6V0A1* | NM_005177 | ATPase, H+ TRANSPORTING LYSOSOMAL V0 SUBUNIT A1; subunit of the vacuolar proton pump. ATP-driven proton pumps associated with clathrin-coated vesicles and synaptic vesicles are a group of polypeptides involved in basic cellular processes through acidification of intracellular organelles. These functions include intracellular targeting of enzymes to lysosomes and secretory granules, and receptor-ligand dissociation in receptor-mediated endocytosis. |
| *CAMK1* | NM_003656 | CALCIUM/CALMODULIN-DEPENDENT PROTEIN KINASE I; subfamily of the serine/threonine protein kinase family. |
| *CAV1* | NM_001753 | CAVEOLIN 1; main component of caveolae membranes. |
| *CAV2* | NM_198212 | CAVEOLIN 2; protein related to caveolin-1, and similar to it in most respects; localizes to caveolae. |
| *CAV3* | NM_001234 | CAVEOLIN 3; muscle-specific form of the caveolin protein family. |
| *CBL* | NM_005188 | CAS-BR-M MURINE ECOTROPIC RETROVIRAL TRANSFORMING SEQUENCE HOMOLOG; functions as a negative regulator of several receptor protein tyrosine kinase signaling pathways and as an adaptor protein in tyrosine phosphorylation-dependent signaling. |
| *CDC42* | NM_044472 | CELL DIVISION CYCLE 42; GTP-binding protein; localizes to the Golgi apparatus of mammalian cells. |
| *CLTA* | NM_001833 | CLATHRIN, LIGHT POLYPEPTIDE A; there are 2 major classes of clathrin light chains, referred to as light chain A (LCA, encoded by the CLTA gene) and LCB (encoded by CTLB). |
| *CLTC* | NM_004859 | CLATHRIN, HEAVY POLYPEPTIDE; clathrin molecules have a triskelion structure composed of 3 non-covalently bound heavy chains (CLTC) and 3 light chains. |
| *DIAPH1* | NM_005219 | DIAPHANOUS 1, DROSOPHILA HOMOLOG; regulates actin polymerization. |
| *DNM1* | NM_004408 | DYNAMIN 1; member of a subfamily of GTP-binding proteins that share considerable sequence homology in their N-terminal domains, but more limited homology in their C-terminal regions. Functions in receptor-mediated endocytosis and is required at an intermediate stage of coated vesicle formation. Believed to assemble around the necks of clathrin-coated pits and assist in pinching vesicles from the plasma membrane. |
| *DNM2* | NM_001005362 | DYNAMIN 2; functions similarly to DNM1; regulates actin reorganization at the immunologic synapse and links to VAV1 and its downstream signaling pathways after TCR engagement. |
| *EFS* | NM_032459 | EMBRYONAL FYN-ASSOCIATED SUBSTRATE; docking protein which plays a central coordinating role for tyrosine kinase-based signaling related to cell adhesion. May serve as an activator of SRC and a downstream effector. Interacts with the SH3 domain of FYN and with CRK, SRC, and YES. |
| *ELKS* | NM_178038 | RAB6-INTERACTING PROTEIN 2; essential regulatory subunit of the IKK complex. Silencing ELKS expression by RNA interference blocks induced expression of NFκB target genes, including the NFκB inhibitor IKBA and proinflammatory genes like cyclooxygenase-2 and interleukin-8. |
| *FYN* | NM_153048 | FYN ONCOGENE RELATED TO SRC, FGR, YES; member of the tyrosine kinase oncogene family; a non-receptor tyrosine protein kinase that plays a role in many biological processes, including regulation of cell growth and survival, cell adhesion, integrin-mediated signaling, cytoskeletal remodeling, cell motility, immune response, and axon guidance. |
| *GIT1* | NM_014030 | G PROTEIN-COUPLED RECEPTOR KINASE-INTERACTING PROTEIN 1; the paxillin-binding domain of GIT1 is required to recruit GIT1 to focal adhesions and the leading edge of the lamellipodia. The central ankyrin repeats and the PIX-binding domain are required to target GIT1 to the cytoplasmic complexes. Expression of GIT1 or its C-terminal paxillin-binding domain increases the rate of migration and the size and frequency of protrusions; PAK association is necessary for this effect. |
| *GRB2* | NM_203506 | GROWTH FACTOR RECEPTOR-BOUND PROTEIN 2; mediates growth factor-induced activation of RAS; implicated in growth factor regulation of the cytoskeleton and DNA synthesis. |
| *HIP1* | NM_005338 | HUNTINGTIN-INTERACTING PROTEIN 1; membrane-associated protein that co-localizes with huntingtin and shares sequence homology and biochemical characteristics with Sla2p, a protein essential for function of *Saccharomyces cerevisiae* cytoskeleton; an endocytic protein, the structural integrity of which may be crucial for maintenance of normal vesicle size *in vivo*. |
| *ITSN1* | NM_001001132 | INTERSECTIN 1; evolutionarily conserved multi-domain protein that functions in clathrin-associated endocytosis and as a mediator of MAPK signaling pathways. |
| *LIMK1* | NM_016735 | LIM DOMAIN KINASE 1; acts in the nucleus to suppress RAC/CDC42 signaling to cyclin D1. LIM kinase is phosphorylated and activated by ROCK, a downstream effector of Rho, and, in turn, phosphorylates cofilin, inhibiting its actin-depolymerizing activity. |
| *MAP4K2* | NM_004579 | MITOGEN-ACTIVATED PROTEIN KINASE KINASE KINASE KINASE 2; GC kinase that specifically activates the SAPK pathway. May play a role in the regulation of vesicle targeting or fusion. |
| *MAPK8IP3* | NM_033392 | MITOGEN-ACTIVATED PROTEIN KINASE 8-INTERACTING PROTEIN 3; a member of a novel class of putative MAPK scaffold proteins that may regulate signal transduction by the JNK pathway. |
| *NEDD4* | NM_006154 | NEURAL PRECURSOR CELL EXPRESSED, DEVELOPMENTALLY DOWNREGULATED 4; WW domains SCNN1A, SCNN1B, and SCNN1G of NEDD4 bind with strong affinity to all 3 subunits of the epithelial sodium channel (ENaC). Both NEDD4 and the related gene KIAA0439 (NEDD4L) may play a role in the regulation of ENaC function. |
| *NSF* | NM_006178 | N-ETHYLMALEIMIDE-SENSITIVE FACTOR; member of the ATPases associated with diverse cellular activities (AAA) gene family involved in vesicular transport. |
| *PDCD6I* | NM_013374 | PROGRAMMED CELL DEATH 6-INTERACTING PROTEIN; also designated ALG-2-interacting protein (Alix); interacts with the endosomal sorting complexes required for transport (ESCRT) proteins TSG101 and CHMP4. Involved in the sorting of cargo proteins of the multivesicular body for incorporation into vesicles. |
| *PICALM* | NM_001008660 | PHOSPHATIDYLINOSITOL-BINDING CLATHRIN ASSEMBLY PROTEIN; very high homology to the murine clathrin assembly protein Ap3. |
| *PIK3C2G* | NM_004570 | PHOSPHATIDYLINOSITOL 3-KINASE, CLASS 2, γ; phosphoinositide 3-kinases (PI3Ks), including PIK3C2G, regulate vesicular transport processes like cargo sorting by phosphorylating the hydroxyl group at the D-3 position of the inositol ring of phosphoinositides. |
| *PIK3CG* | NM_002649 | PHOSPHATIDYLINOSITOL 3-KINASE, CATALYTIC, γ; PI3K plays a pivotal role in the regulation of cytotoxicity in natural killer cells. |
| *RAB1A* | NM_004161 | RAS-ASSOCIATED PROTEIN RAB1A; regulates vesicular protein transport from the endoplasmic reticulum (ER) to the Golgi compartment and on to the cell surface. Plays a role in microtubule-dependent protein transport by early endosomes. |
| *RAB2* | NM_002865 | RAS-ASSOCIATED PROTEIN RAB2; required for protein transport from the ER to the Golgi complex. |
| *RAB5A* | NM_004162 | RAS-ASSOCIATED PROTEIN RAB5A; RAB5 is a rate-limiting component of the machinery regulating the kinetics of membrane traffic in the early endocytic pathway. |
| *RAB5C* | NM_004583 | RAS-ASSOCIATED PROTEIN RAB5C; shares 86% identity with RAB5A (179512) and RAB5B (179514) and contains the conserved GTP-binding site characteristic of RAS proteins. |
| *RAB7L1* | NM_003929 | RAB7-LIKE 1; shows sequence similarity to RAB7 and contains the 4 GTP-binding domains that are conserved in RAS proteins, although 2 of its domains have amino acid substitutions at conserved sites. |
| *RAB8A* | NM_005370 | RAS-ASSOCIATED PROTEIN RAB8A; responsible for the localization of apical proteins in intestinal epithelial cells. |
| *RAC1* | NM_006908 | RAS-RELATED C3 BOTULINUM TOXIN SUBSTRATE 1; RAS superfamily is involved in actin polymerization, and plays an important role in phagocytosis/macropinocytosis. |
| *ROCK1* | NM_005406 | RHO-ASSOCIATED COILED-COIL-CONTAINING PROTEIN KINASE 1; protein serine/threonine kinase activated by binding to the GTP-bound form of Rho. |
| *SYT1* | NM_005639 | SYNAPTOTAGMIN I; integral membrane protein in synaptic vesicles thought to serve as Ca^2+^ sensors in the process of vesicular trafficking and exocytosis. Calcium binding to synaptotagmin I participates in triggering neurotransmitter release at the synapse. |
| *TNIK* | NM_015028 | TRAF2- AND NCK-INTERACTING KINASE; one of the germinal center kinases (GCKs), which are characterized by an N-terminal kinase domain and a C-terminal GCK domain that serves a regulatory function. May be involved in cytoskeleton regulation, and is a specific effector of RAP2 in regulating the actin cytoskeleton |
| *TSG101* | NM_006292 | TUMOR SUSCEPTIBILITY GENE 101 PROTEIN; component of the ESCRTI complex. Binds to ubiquitinated cargo proteins and is required for the sorting of endocytic ubiquitinated cargo into multivesicular bodies. Mediates the association between the ESCRT0 and ESCRTI complex. |
| *VPS4A* | NM_013245 | VACUOLOR PROTEIN SORTING 4 HOMOLOG A**;** involved in late steps of the endosomal multivesicular bodies pathway. Recognizes membrane-associated ESCRTIII assemblies and catalyzes their disassembly, possibly in combination with membrane fission. Redistributes ESCRTIII components to the cytoplasm for further rounds of multivesicular body sorting. |
| *WASF2* | NM_006990 | WISKOTT-ALDRICH SYNDROME PROTEIN FAMILY, MEMBER 2; a downstream effector molecule involved in transmitting signals from tyrosine kinase receptors and small GTPases to the actin cytoskeleton. |
| *WASF3* | NM_006646 | WISKOTT-ALDRICH SYNDROME PROTEIN FAMILY, MEMBER 3; WASF3 expression is strongest in brain. WASF3 binds actin through its C-terminal verprolin homology domain. |
