# Supplementary material for: Endocytic Pathways Used by Andes Virus to Enter Primary Human Lung Endothelial Cells
Source: PLoS One. 2016 Oct 25;11(10):e0164768. doi: 10.1371/journal.pone.0164768 (PMC5079659; doi:10.1371/journal.pone.0164768)
Supplement: S3 Table — (DOCX) [file pone.0164768.s006.docx]

**S3 Table. Viral protein levels and ANDV release determined by western blotting and plaque assays, respectively^a^.**

| **Gene Symbol** | **% Gene Product ↓** | | **% ANDV N↓** | | **% ANDV G↓** | | **% Virus Release↓** | |
| --- | --- | --- | --- | --- | --- | --- | --- | --- |
|  | **24 h** | **48 h** | **24 h** | **48 h** | **24 h** | **48 h** | **24 h** | **48 h** |
| **DNM2** | n/a | 80 | 96 | 93 | 100 | 100 | n/a | 73 |
| **CLTC** | n/a | 92 | 100 | 100 | 100 | 100 | n/a | 96 |
| **AP2M1** | 65 | 65 | 7 | 0 | -88 | -303 | 63 | 11 |
| **CAV1** | 57 | 59 | 12 | 3 | 12 | -158 | 69 | 5 |
| **CDC42** | 66 | 91 | -15 | 42 | 16 | 28 | 53 | -21 |
| **ARF6** | 100 | 100 | 24 | -11 | -13 | -324 | 75 | 26 |
| **ARF1** | n/a | 89 | n/a | 70 | n/a | 99 | n/a | 34 |
| **AP1G1** | 92 | 64 | 72 | 78 | 73 | 25 | 80 | 40 |
| **NSF** | 79 | 100 | 59 | 0 | 38 | 43 | 20 | 53 |
| **RAB5C** | 93 | 89 | 41 | 52 | 41 | 49 | 49 | 23 |
| **TSG101** | 45 | 78 | -56 | -16 | -54 | -44 | 50 | 46 |

^a^ % Values were calculated relative to non-targeting siRNA control. Negative values represent % increase rather than knockdown.
